# Evaluation of monocular and binocular contrast perception on virtual reality head-mounted displays

**DOI:** 10.1117/1.JMI.11.6.062605

**Published:** 2024-09-14

**Authors:** Khushi Bhansali, Miguel A. Lago, Ryan Beams, Chumin Zhao

**Affiliations:** US Food and Drug Administration, Center for Devices and Radiological Health, Silver Spring, Maryland, United States

**Keywords:** virtual reality, binocular vision, image quality assessment, contrast, contrast perception

## Abstract

**Purpose:**

Visualization of medical images on a virtual reality (VR) head-mounted display (HMD) requires binocular fusion of a stereoscopic pair of graphical views. However, current image quality assessment on VR HMDs for medical applications has been primarily limited to time-consuming monocular optical bench measurement on a single eyepiece.

**Approach:**

As an alternative to optical bench measurement to quantify the image quality on VR HMDs, we developed a WebXR test platform to perform contrast perceptual experiments that can be used for binocular image quality assessment. We obtained monocular and binocular contrast sensitivity responses (CSRs) from participants on a Meta Quest 2 VR HMD using varied interpupillary distance (IPD) configurations.

**Results:**

The perceptual result shows that contrast perception on VR HMDs is primarily affected by optical aberration of the VR HMD. As a result, monocular CSR degrades at a high spatial frequency greater than 4 cycles per degree when gazing at the periphery of the display field of view, especially for mismatched IPD settings consistent with optical bench measurements. On the contrary, binocular contrast perception is dominated by the monocular view with superior image quality measured by the contrast.

**Conclusions:**

We developed a test platform to investigate monocular and binocular contrast perception by performing perceptual experiments. The test method can be used to evaluate monocular and/or binocular image quality on VR HMDs for potential medical applications without extensive optical bench measurements.

## 1 Introduction

Rapid advancement in virtual reality (VR) technology has broadened its implementation beyond entertainment^1^ toward education^2^ and a wide variety of medical applications.^3^^–^^5^ Clinically, VR has been investigated for the visualization of two-dimensional (2D) and three-dimensional (3D) medical images^6^^,^^7^ and preoperative surgical planning^8^ in an immersive environment. It has also shown potential for patient-facing vision therapy such as amblyopia treatment^9^^–^^11^ and pain management.^12^ Technically, to create an immersive user experience, VR head-mounted displays (HMDs) present a pair of virtual images on two eyepieces generalizing a stereoscopic visualization of a virtual scene. Unlike on conventional flat-panel displays, the display hardware and optics are independent of two eyepieces. On the other hand, image rendering and processing techniques also interact interocularly on the display pipeline, followed by binocular fusion by the user or the patient. Therefore, visual experience and clinical effectiveness on VR HMDs are affected by both monocular and binocular image quality.

**Fig. 1:**
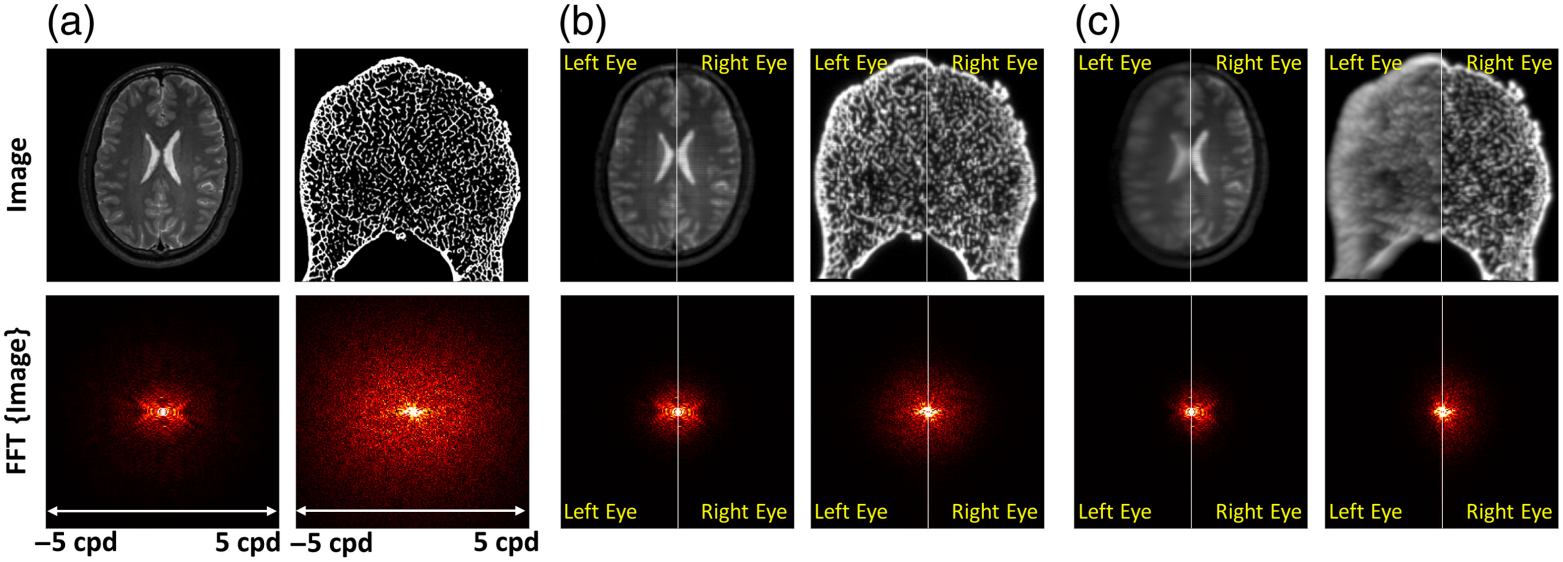
(a) Ground truth: example medical images of a brain MR and a segmented vertebra microstructure (top row) and the images in the Fourier spatial frequency domain (bottom row). (b) Central views, IPD aligned: monocular visualization of the two medical images in panel (a) on a VR HMD placed at the center of the display FoV with the camera position aligned with the HMD IPD setting—left half, left eye visualization; right half, right eye visualization. (c) Peripheral views, IPD misaligned: the same as panel (b) but with the medical images placed at the periphery (−9-deg azimuth angle) of the display FoV and with a 1-cm IPD mismatch between the camera and HMD (0.5-cm translation of the camera toward the temporal direction on either eye).

On each HMD eyepiece, technical challenges in optics, display, and sensor technologies limit the monocular image quality on VR HMDs in both spatial and temporal domains.^13^ Specifically, the pixel resolution limit of VR HMDs is typically ∼10 to 20 cycles per degree (cpd), which is lower than the human vision limit of ∼60  cpd. The image resolution can also be affected by the software and rendering techniques such as anti-aliasing,^14^ image warping,^15^ foveated rendering,^16^ and trade-offs between spatial resolution and latency.^17^ In addition, optical aberration by the VR lenses can substantially degrade the image contrast and resolution on each eyepiece, especially at the periphery of the display field of view (FoV) when the optical axes of the human eye and VR lens are misaligned.^18^^,^^19^ For instance, as illustrated in Fig. 1(a), two example medical images, i.e., axial slices of a brain MR image (left) and a segmented vertebra microstructure image (right), are rendered on a VR HMD at the center of the display FoV shown in Fig. 1(b). These two images are transformed into the Fourier domain as shown in the bottom row of Fig. 1. The vertebra microstructure contains more high-spatial-frequency content. However, image quality degradation through the VR display pipeline including the rendering engine, display pixelation, and optical aberration by the VR lens dramatically suppresses the spatial resolution (see the Fourier-domain vertebra image in Fig. 1(b) captured by a front aperture camera). Note that this is under the condition that the entrance pupil of the camera was placed at the eye point of the HMD and that the image was centered to ensure optimal image quality with the user’s interpupillary distance (IPD) or camera’s entrance pupil position matching the physical IPD setting of the VR HMD.

Although most VR HMDs enable physical IPD adjustment, the adjustable IPD range may be limited, which is challenging for users or patients with small (e.g., pediatric patients) or large IPDs. Monocular image quality can be further contaminated if the IPD of the user does not match that of the HMD. For instance, as shown in Fig. 1(c), the camera was shifted laterally by 5 mm toward the temporal direction, emulating an IPD mismatch of 1 cm. At the same time, the medical images were placed at the periphery of the display FoV (at −9  deg azimuth angle toward the left-hand side of the user). In this case, the optical axes of the lens and the camera are misaligned resulting in a more substantial reduction in spatial resolution as shown in Fig. 1(c), in comparison with the central views with the correct IPD setting in Fig. 1(b). It is clearly visualized that image quality is not identical on both eyepieces. More specifically, in this setup with a temporal direction eye rotation plus pupil position translation, image resolution degradation due to optical aberration is further amplified on the left eyepiece [see the comparison between the left and right eye visualizations shown as the left half and right half images in Fig. 1(c)] leading to binocular image quality discrepancy. This is not only shown in the high-spatial-frequency content such as bone microstructure but also affects the low-contrast content perception such as the brain MR image as an example. Image quality degradation and binocular inconsistency can potentially affect the clinical effectiveness of VR for medical applications. However, the evaluation of binocular image quality on VR HMDs is technically challenging without established methods or standards.

Recent work has evaluated the impact of binocular inconsistency on VR HMDs for applications such as gaming.^20^^,^^21^ However, the current evaluation of image quality on VR HMDs is still generally based on monocular optical bench measurements with test methods established in the International Electrotechnical Commission (IEC) 63145-20 standard^22^ and Information Display Measurements Standard.^23^ It remains uncertain whether these measurements adequately capture the binocular image quality experienced by users in medical applications. In vision science, a number of binocular summation models have been studied by fitting the contrast perception and phase data from perceptual experiments.^24^^–^^30^ However, it is technically challenging to combine the complicated binocular model (except for the oversimplified “$\sqrt{2}$” model^31^) primarily in the image domain with optical bench testing results. In addition, specific requirements on the light measuring devices (LMDs) and bench setup have been recommended in the standards to emulate the eye anatomy and rotation mechanism to investigate gaze-dependent monocular image quality on VR HMDs. Unfortunately, the experimental setup is nontrivial to enable an eye rotation geometry, e.g., 5 degrees of freedom (DoFs) translational and rotational stages are recommended in the IEC 63145-20-10 standard.^32^ At the same time, the LMD should be compact to fit the bench setup and carefully calibrated yielding accurate luminance and spatial measurement results.^33^

In this study, as an alternative to optical bench testing, we present a software platform to evaluate binocular image quality on VR HMDs across the display FoV by performing human contrast perception experiments to measure the contrast sensitivity response (CSR). The perceptual experiments aim to bypass the limitations of bench testing for more efficient image quality evaluation without advanced lab equipment. We compare the monocular and binocular contrast detection results to emphasize the image quality discrepancies interocularly and between monocular and binocular perception using different IPD configurations.

## 2 Methods

### 2.1 Head-Mounted Display

A Meta Quest 2 HMD was used for perceptual experiments to measure the monocular and binocular CSRs from the participants. The Meta Quest 2 HMD is based on a fast-switching liquid crystal display (LCD) backplane with a pair of Fresnel lenses. The pixel resolution of the display backplane is 1832 (horizontal) × 1920 (vertical) pixels per eye, refreshing at 90 Hz. The Fresnel lenses magnify the virtual images yielding a horizontal FoV of ∼90  deg. It offers three physical IPD settings, i.e., 58 mm (small), 63 mm (nominal), and 68 mm (large), providing users with options to customize the HMD IPD for optimal viewing comfort and visual performance. The Meta Quest 2 HMD was selected to validate the test platform as described in the section below. There are many different optical and display designs for VR HMDs such as using pancake lenses or microdisplays that may vary the perceptual test results. The goal of this paper is to develop the test method instead of comparing the perceptual experimental results across various HMDs and display technologies.

### 2.2 WebXR Target Detection Platform

We developed a WebXR platform to perform perceptual experiments by determining the CSR defined as the reciprocal of threshold contrast ($C_{th}$) that a participant can detect on a VR HMD. Note that the CSR measured in this study is different from the contrast sensitivity function (CSF) of the naked human eyes without HMD modulation.^34^ In other words, CSR measures the CSR that incorporates the HMD optical aberration, whereas CSF describes the human contrast perception capability without the HMD. WebXR is an application programming interface for developing and hosting VR and AR web-based applications on compatible mixed-reality headsets. The WebXR application was built using the A-Frame library and was executed on an Oculus Browser that displayed Gabor stimuli for detection.

The Gabor target is a 2D sinusoidal pattern with predefined contrast ($C_{\mathrm{GL}}$), whose dimension is determined by the standard deviation of a Gaussian bracket (σ) and spatial frequency (f). The Gabor target centered at location $\left(x_{0},y_{0}\right)$ can be illustrated as

$$G(x,y)=C_{\mathrm{GL}}\cdot I_{b}\cdot \cos(2\pi fr)\cdot \exp(-\frac{(x-x_{0}{)}^{2}+(y-y_{0}{)}^{2}}{2\sigma^{2}})+I_{b},$$ (1)

where $I_{b}$ and $C_{\mathrm{GL}}$ are the background display gray level (GL) and modulation of the Gabor target determined by the display GL, respectively, and the exponential term describes the Gaussian envelope. r is the radial distance with respect to the center of the stimuli that is given by

$$r=(x-x_{0})\cdot \cos\;\theta +(y-y_{0})\cdot \sin\;\theta ,$$ (2)

where θ is associated with the angular location of the stimuli. The standard deviation (σ) of the Gaussian bracket is preset to ∼0.5  deg (radius of ∼1  deg). The spatial frequency f of the Gabor target varied from 0.52 to 5.72 cycles per degree covering the most sensitive frequency range of the human eyes.

**Fig. 2:**
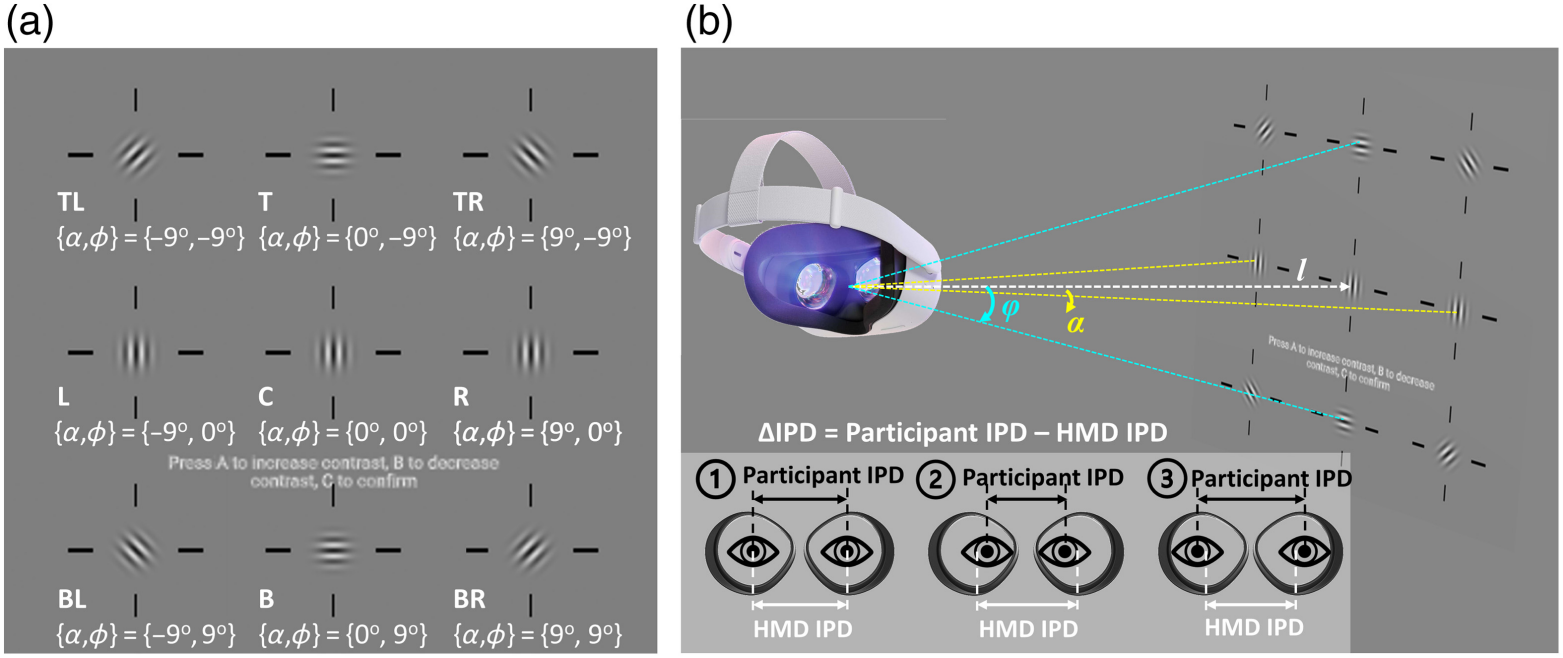
(a) Illustrations of the Gabor target placed at nine locations across the FoV with the orientation of the sinusoidal pattern aligned circularly (graph for illustration only, dimension not scaled). (b) Visual illustration of the perceptual experiments and grouping of participants, namely, group 1: participant IPD≈HMD IPD, group 2: participant IPD<HMD IPD, and group 3: participant IPD>HMD IPD.

As shown in Fig. 2(a), the Gabor target was placed at nine locations across the FoV following the IEC 63145-20-20 standards^22^ at a long distance of 150 m away such that the target aligns with the optical axis of eyepiece lenses when placed at the center (α and ϕ are zero). The target angular dimension is ∼1  deg in diameter. We admit that the long view distance may cause visual discomfort due to the vergence–accommodation conflict.^35^ The impact of VR visual distance on contrast sensitivity should be evaluated in future work. Table 1 summarizes the 3D coordinates of the target at various locations. For example, moving from the center to the right, the target was laterally translated by 25 m, i.e., $(x_{0},y_{0},z)=(25,0,-150)\;m$ corresponding to an azimuth angular rotation of ∼9  deg, i.e., (α,ϕ)=(9  deg,0  deg). At the top right position, an additional 25-m translation was added on $y_{0}$, yielding azimuth and elevation rotations of (α,ϕ) of (∼9  deg,−9  deg), corresponding to an angle of ∼13.3  deg diagonally. This angle will ensure that the target is within the participant’s FoV for all IPD conditions in the perceptual experiments to be described in Sec. 2.3. At each location, the orientation of the target was modified such that the sinusoidal contrast pattern is always circular (or perpendicular to the radial direction) with respect to the target location. It has been shown that the circular orientation of the pattern is suitable to capture the contrast degradation by optical aberration.^19^

**Table 1:** Physical parameters of the Gabor target.

Parameters	Description	Value	Unit
$\left(x_{0},y_{0},z\right)$	3D coordinates of the target at the center	(0, 0, −150)	m
	At the top and bottom	(0, ±25, −150)	m
	At the left and right	(±25, 0, −150)	m
	At the four corners	(±25, ±25, −150)	m
(α,ϕ)	Angular position of the target at the center	(0, 0)	deg
	At the top and bottom	(0, ±9)	deg
	At the left and right	(±9, 0)	deg
	At the four corners	(±9, ±9)	deg
$C_{\mathrm{GL}}$	Contrast of the target	0.004 to 1	—
σ	Standard deviation of the Gaussian bracket	1.3 (0.5)	m (deg)
f	Spatial frequency of the Gabor target	0.52 to 5.72	Cycles per degree

### 2.3 Perceptual Experiments

The Food and Drug Administration (FDA) Institutional Review Board (IRB) reviewed and approved the study protocol (IRB 2022-CDRH-038) and that all human participant studies were conducted under the approved protocol. We recruited nine human participants (with ages between 21 and 47 years, average age of 27.7, three females, and six males) with normal or corrected to normal vision by wearing refractive glasses in the HMD to participate in the perceptual experiments. Therefore, the potential impacts of visual acuity, astigmatism, and age (presbyopia) on the perceptual experimental results are minimized. In addition, the evaluated spatial frequency range is up to 5.72 cycles per degree, which is primarily limited by the HMD resolution. Such spatial frequencies are much lower than the human visual system capability up to ∼60 cycles per degree. In this case, we ensure that the impact of visual acuity variation among the participants should not substantially affect the test results. The IPD of the participants was recorded. Among the nine participants, three have small IPDs of less than 61 (mean IPD of 58.3 mm) corresponding to the small HMD IPD setting of 58 mm. Three participants with large IPDs beyond 66 mm (mean IPD of 70.3 mm) agree with the 68-mm IPD setting of the HMD. The remaining three participants have IPDs within the range of 61 to 66 mm (mean IPD of 63.3 mm) in alignment with the nominal HMD IPD setting of 63 mm. Perceptual experiments were conducted in three groups on each combination of participant IPD and HMD hardware setting:

Group 1: participant IPD≈HMD IPD. All nine participants participated in the group 1 experiments by properly adjusting the HMD IPD setting to match their own IPD.
Group 2: participant IPD<HMD IPD. The three participants with small IPD of less than 61 mm (mean IPD of 58.3 mm) participated in this set of experiments by setting the HMD IPD to a maximum of 68 mm.
Group 3: participant IPD>HMD IPD. The three participants with large IPD greater than 66 mm (mean IPD of 70.3 mm) participated in this set of experiments by setting the HMD IPD to a minimum of 58 mm.

Each human observer experiment contains 45 trials (nine target locations × five Gabor spatial frequencies). Prior to the experiment, the physical IPD setting of the HMD was adjusted. During each trial, the participant was instructed to observe the threshold contrast of the target shown on the Quest 2 HMD by adjusting the contrast of the Gabor target using a controller or a Bluetooth keyboard with a minimum adjustable contrast of 0.004. Once the participant determined the threshold contrast ($C_{th}$), the contrast sensitivity, as the reciprocal of the measured threshold contrast, at a specified location and spatial frequency was recorded. For each participant, the above experiment was repeated three times binocularly with both eyes open and monocularly on only the left or right eye by wearing an eye patch on the other eye underneath the headset. The result of each experiment was saved into a JavaScript Object Notation (JSON) file on the headset. We extracted the files and then obtained the HMD-modulated CSR over the spatial frequencies.

Although the user-controlled determination of threshold contrast features fast experiments for the evaluation of multiple spatial frequencies and IPD settings, it introduces potential bias and random error from the participant. In future work, a stair-step procedure with a binary forced choice task (i.e., a yes–no detection task) can be investigated to eliminate potential bias from human participants.^36^

### 2.4 Impact of Display Luminance Response on Contrast Perception

As illustrated in Sec. 2.2, the WebXR engine defines the input contrast ($C_{\mathrm{GL}}$) based on the digital content (i.e., display GLs)

$$C_{\mathrm{GL}}=\frac{I_{h}-I_{l}}{I_{h}+I_{l}}=\frac{\left(I_{b}+\Delta )-(I_{b}-\Delta \right)}{\left(I_{b}+\Delta )+(I_{b}-\Delta \right)}=\frac{\Delta }{I_{b}},$$ (3)

where $I_{h}$ and $I_{l}$ are the peak (bright lines) and minimum (dark lines) GLs of the Gabor target. If we define Δ as the amplitude of the signal (sinusoidal wave) in GLs, then $I_{h}=I_{b}+\Delta$ and $I_{l}=I_{b}-\Delta$.

**Fig. 3:**
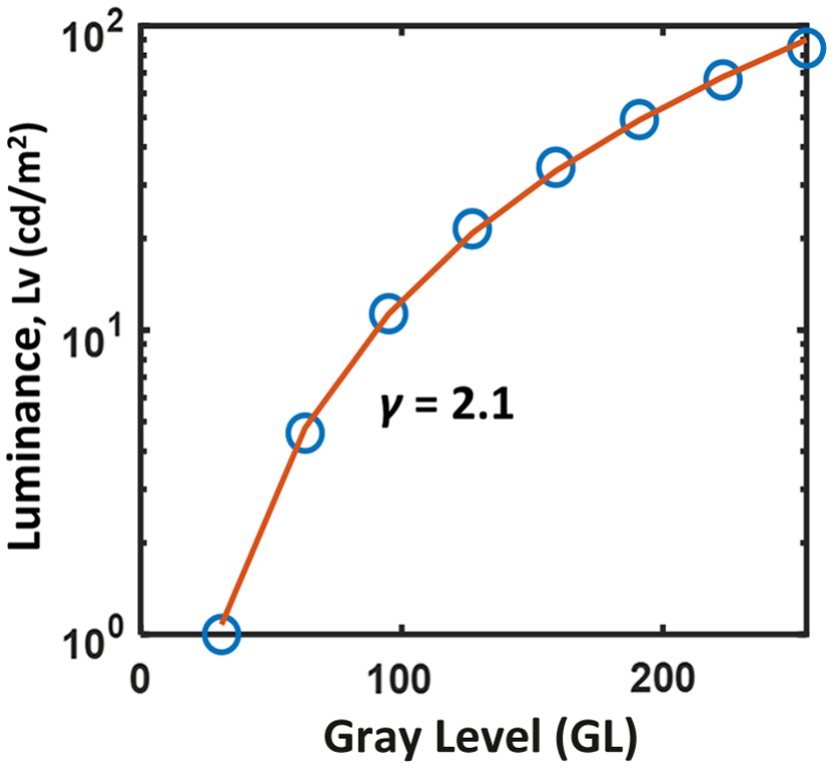
Display luminance response of the evaluated Meta Quest 2 HMD.

However, it should be clarified that the input contrast for the display differs from the perceptual contrast for an observer. The perceptual contrast ($C_{\mathrm{Lv}}$) is generally determined by the luminance difference of the target, which can be expressed as

$$C_{\mathrm{Lv}}=\frac{L_{h}-L_{l}}{L_{h}+L_{l}},$$ (4)

where $L_{h}$ and $L_{l}$ are the peak and minimum luminance of the Gabor target. $C_{\mathrm{GL}}$ equals to $C_{\mathrm{Lv}}$ if the display luminance is linear to the display GL. Otherwise, the display luminance response, also known as the gamma curve, needs to be taken into account when computing the perceptual contrast. If the dark luminance of the display is ignored, a simplified display luminance response can be expressed as

$$L_{n}=L_{\max}{\left(I_{n}/I_{\max}\right)}^{\gamma },$$ (5)

where $L_{n}$ is the display luminance at GL $I_{n}$. $L_{\max}$ and $I_{\max}$ are the maximum luminance and GL of the display. Figure 3 shows the measured display luminance response of the evaluated Meta Quest 2 HMD in the logarithm scale with display peak luminance of $88\;\mathrm{cd}/m^{2}$ and display gamma γ extracted as ∼2.1 by fitting the display luminance at various GLs. The luminance measurement was performed using a calibrated imaging photometer (LMK 6, TechnoTeam, Ilmenau, Germany).

The display luminance response can be used to convert the display GLs to luminance. Therefore, by substituting Eqs. (3) and (5) into Eq. (4), the input contrast can be converted to perceptual contrast by

$$C_{Lv}=\frac{{\left(I_{b}+\Delta \right)}^{\gamma }-{\left(I_{b}-\Delta \right)}^{\gamma }}{{\left(I_{b}+\Delta \right)}^{\gamma }+{\left(I_{b}-\Delta \right)}^{\gamma }}=\frac{{\left(1+C_{\mathrm{GL}}\right)}^{\gamma }-{\left(1-C_{\mathrm{GL}}\right)}^{\gamma }}{{\left(1+C_{\mathrm{GL}}\right)}^{\gamma }+{\left(1-C_{\mathrm{GL}}\right)}^{\gamma }}.$$ (6)

If the detected threshold contrast is small, i.e., for a small Δ

$$C_{\mathrm{Lv},\Delta \approx 0}\approx \frac{\left(1+\gamma C_{\mathrm{GL}})-(1-\gamma C_{\mathrm{GL}}\right)}{\left(1+\gamma C_{\mathrm{GL}})+(1-\gamma C_{\mathrm{GL}}\right)}=\gamma C_{\mathrm{GL}}.$$ (7)

On the other hand, if the image quality is poor leading to a large threshold contrast, i.e., $C_{\mathrm{GL}}\approx 1$ or $\Delta \approx I_{b}$

$$C_{Lv,\Delta \approx I_{b}}\approx C_{\mathrm{GL}}\approx 1.$$ (8)

It is indicated that the nonlinear display luminance response leads to different threshold contrast computed as the GL ($C_{\mathrm{GL}}$) and luminance modulation ($C_{\mathrm{Lv}}$). Therefore, we distinguish CSR computed using input display GL and luminance as ${\mathrm{CSR}}_{\mathrm{GL}}$ and ${\mathrm{CSR}}_{\mathrm{Lv}}$ in the presented results, respectively.

## 3 Results

### 3.1 Monocular and Binocular Contrast Perception for Participants with Different IPDs

#### 3.1.1 Monocular contrast perception

**Fig. 4:**
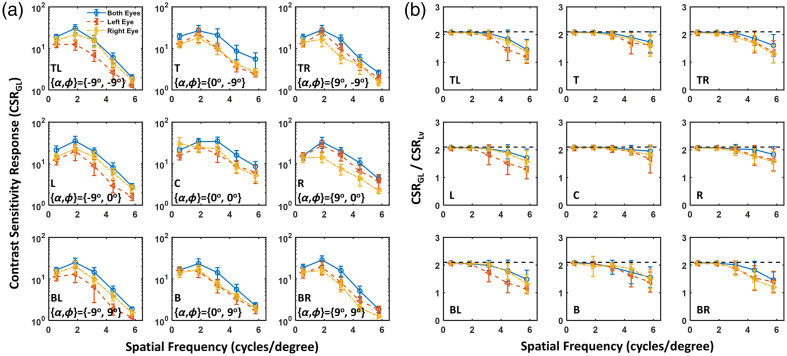
(a) Monocular (red color, left eye; yellow color, right eye) and binocular (blue color, both eyes) ${\mathrm{CSR}}_{\mathrm{GL}}$ for participants in group 1 with the appropriate IPD setting measured at various locations on the Quest 2 HMD. (b) Ratio between ${\mathrm{CSR}}_{\mathrm{GL}}$ and ${\mathrm{CSR}}_{\mathrm{Lv}}$ for the perceptual data shown in panel (a).

**Fig. 5:**
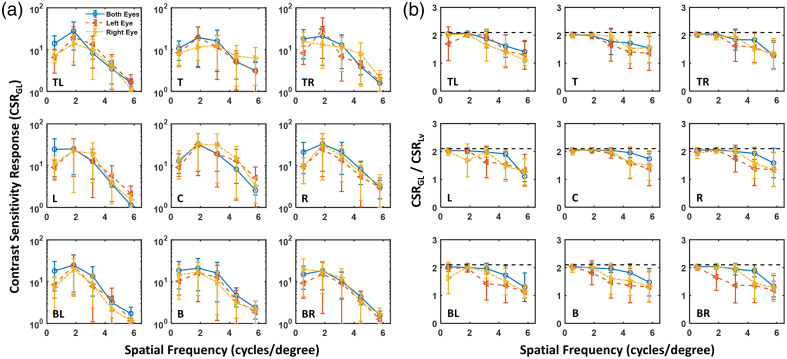
(a) Monocular (red color, left eye; yellow color, right eye) and binocular (blue color, both eyes) contrast sensitivity functions for participants in group 2 with IPD smaller than that of the HMD measured at various locations on the Quest 2 HMD. (b) Ratio between ${\mathrm{CSR}}_{\mathrm{GL}}$ and ${\mathrm{CSR}}_{\mathrm{Lv}}$ for the perceptual data shown in panel (a).

**Fig. 6:**
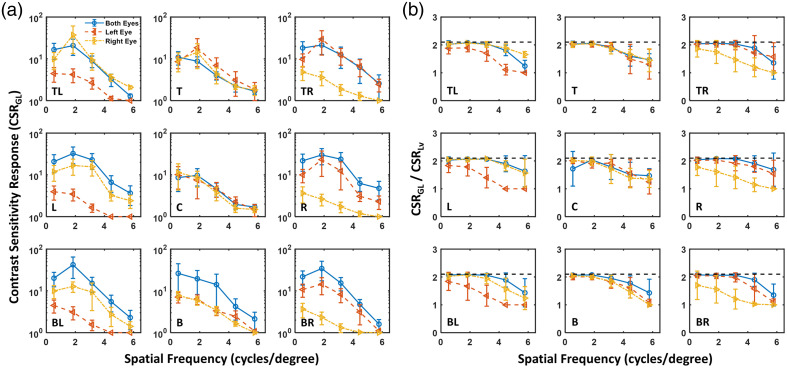
(a) Monocular (red color, left eye; yellow color, right eye) and binocular (blue color, both eyes) contrast sensitivity functions for participants in group 3 with IPD greater than that of the HMD measured at various locations on the Quest 2 HMD. (b) Ratio between ${\mathrm{CSR}}_{\mathrm{GL}}$ and ${\mathrm{CSR}}_{\mathrm{Lv}}$ for the perceptual data shown in panel (a).

Group 1 experiments: First, we focus on monocular contrast sensitivity (see red and yellow lines in Figs. 4–6). For participants using the appropriate IPD configuration on the HMD (group 1 participants), as shown in Fig. 4 (a), ${\mathrm{CSR}}_{\mathrm{GL}}$ is optimized at the center of display FoV, yielding improved contrast perception at spatial frequencies greater than 4 cycles per degree compared with peripheral target locations. It has been reported that optical aberration of the HMD lens degrades the contrast and effective resolution at the periphery of VR display FoV^19^ due to the shift of visual point on an eyepiece (Δd).

Group 2 experiments: IPD misalignment, where the IPD of the participant does not match the physical lens displacement of the HMD, negatively affects the monocular image quality. For participants with smaller IPD than that of the HMD (group 2 participants), Fig. 5(a) shows no obvious difference between nasal (e.g., right eye gazing toward left) and temporal (e.g., right eye gazing at the right) ${\mathrm{CSR}}_{\mathrm{GL}}$ nor major interocular contrast perception difference between the left and right eyes. We suspect this is due to the effect of shifts of visual point and visual axis compensating for each other with details and an experimental validation provided in Sec. 3.3.

Group 3 experiments: Fig. 6(a) shows that interocular ${\mathrm{CSR}}_{\mathrm{GL}}$ variation is substantially pronounced for participants with larger IPD than that of the HMD (group 3 participants). ${\mathrm{CSR}}_{\mathrm{GL}}$ drops dramatically if the eye is rotated in the temporal direction (e.g., the right eye rotates toward the right). As illustrated in Sec. 3.3, this is because both the visual point and visual angle shift favor nasal eye rotation.

#### 3.1.2 Binocular contrast perception

As shown in Figs. 4–6(a) in blue curves, binocular perception on a VR HMD is primarily dominated by the eye with superior contrast sensitivity. This observation is consistent with the finding of interocular contrast difference by Wang et al.^37^ in an augmented reality setup. Binocular image quality on VR HMDs, as measured by the ${\mathrm{CSR}}_{\mathrm{GL}}$, does not always equal to the monocular perception. The difference is particularly enhanced for misaligned IPD between the human participant and HMD optics (e.g., see Fig. 6). A simple “$\sqrt{2}$” model^31^ may be used to compute binocular CSR from monocular perceptual data. However, this model may be oversimplified for the complex human visual system. Recent work has investigated the interocular contrast and color differences in binocular AR displays using binocular summation models in the image domain.^37^^,^^38^

### 3.2 Comparison Between CSR Computed by Input Display Gray Level and Display Luminance

As illustrated in Sec. 2.4, the nonlinear display luminance response results in a difference in threshold contrast computed using display GLs ($C_{\mathrm{GL}}$) and display luminance ($C_{\mathrm{Lv}}$). As shown in Figs. 4–6(b), the ratio of ${\mathrm{CSR}}_{\mathrm{GL}}{/\mathrm{CSR}}_{\mathrm{Lv}}$ equals to $C_{\mathrm{Lv}}{/C}_{\mathrm{GL}}$, which approaches to γ of 2.1 when the threshold contrast is small or for higher CSR values at low spatial frequencies. On the other hand, the ratio drops to 1, indicating ${\mathrm{CSR}}_{\mathrm{GL}}$ approaches ${\mathrm{CSR}}_{\mathrm{Lv}}$ when the threshold contrast is close to 1, i.e., very poor contrast detectability at the high spatial frequencies especially for IPD mismatched conditions. It should be emphasized that the WebXR rendering engine does not involve display luminance calibration. Therefore, the contrast and CSR obtained from the WebXR platform are computed based on the display input GLs. To convert threshold contrast and CSR into the luminance domain, display luminance response as shown in Fig. 3 should be obtained.

### 3.3 Impacts of IPD Misalignment and Eye Rotation Geometry

To better understand the perceptual results shown in Figs. 4–6, we investigate the impact of IPD misalignment and eye rotation geometry. Specifically, we believe two geometrical parameters would affect the image quality on the VR HMD: the spatial variation of the lateral shift of the visual point on the HMD eyepiece denoted as Δd and the angle between the optical axes of the eye and HMD lens defined as Δβ.

**Fig. 7:**
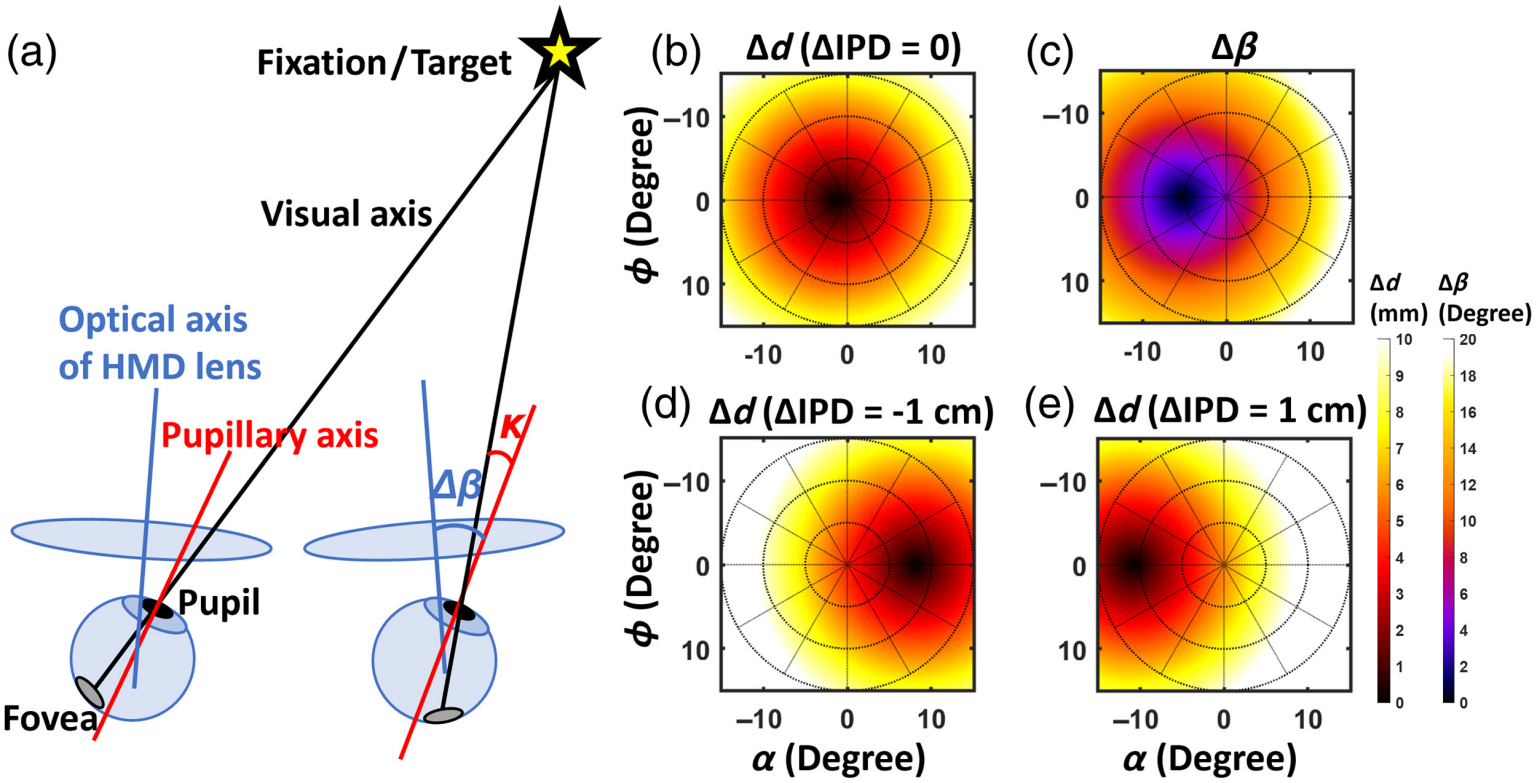
(a) Schematic illustration of the binocular eye rotation geometry with optical axis of the HMD lens (blue color), pupillary axis of the eye (red color), and visual axis (black color) shown. Illustrations of spatial variation of the lateral shift of the visual point on the right eyepiece (Δd) for (b) ΔIPD of 0, (d) −1 and (e) 1 cm, and (c) the angle between the optical axes of the right eye and the right eyepiece lens of the HMD (Δβ). Angle kappa and Δβ are highlighted in panel (a).

For group 1 participants, as illustrated in Fig. 7(a), Δd without IPD misalignment (ΔIPD=0) is approximately angular symmetric on the right eyepiece but increases radially indicating increased aberration. Comparing monocular CSRs between the left and right eyes with appropriate IPD settings, it shows that contrast detection on the Quest 2 HMD slightly favors nasal eye rotation, i.e., the left eye gazing toward the right or the right eye toward the left (see CSR results in Fig. 4).

**Fig. 8:**
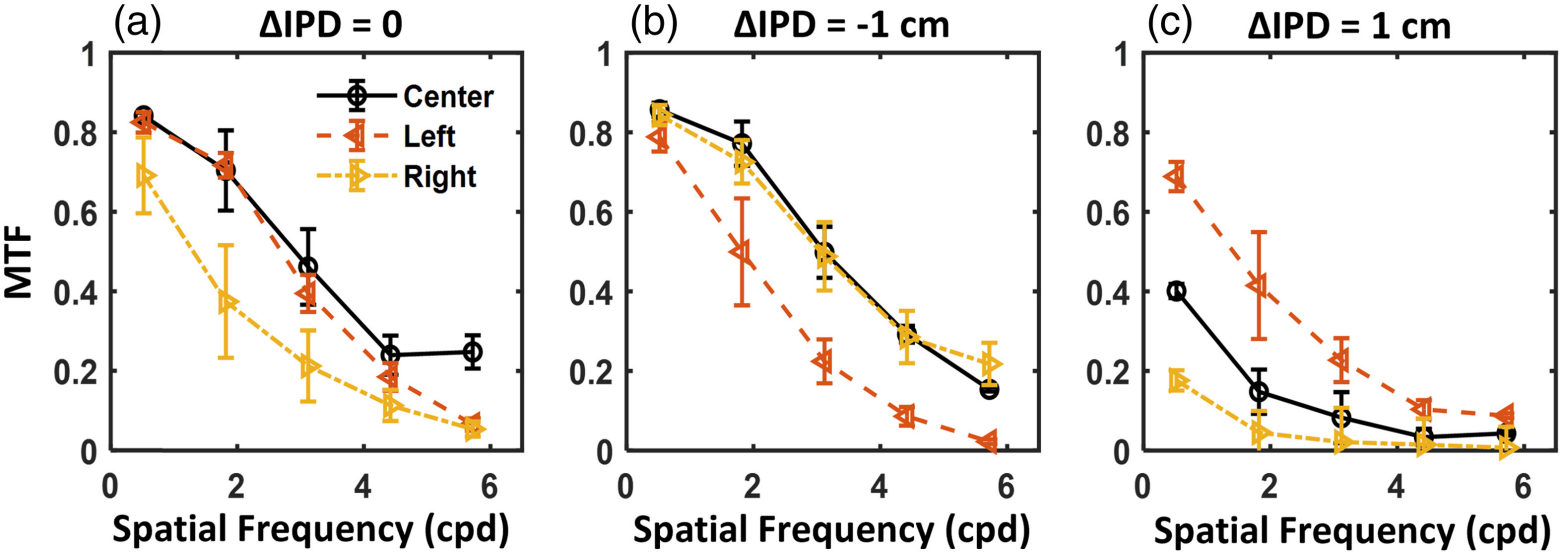
Measured modulation transfer functions on the right eyepiece of the Quest 2 HMD using various IPD settings with ΔIPD of (a) 0, (b) −1, and (c) 1 cm, similar to the configurations of groups 1, 2, and 3 in perceptual experiments. The experimental setup enables an eye rotation geometry with an additional 4-deg rotation to emulate the angle kappa with the Gabor target placed at the same location as the perceptual experiments (i.e., left α=−9  deg, center and right α=9  deg).

We suspect that the variation of monocular vision is mainly associated with the angular rotation between the optical axes of the eye (involving angle kappa of the human visual system^39^^,^^40^) and HMD eyepiece lens (see Δβ shown in Fig. 7(b) for the right eye). As illustrated in Fig. 7(a), angle kappa is defined as the angle between the pupillary axis (i.e., optical axis of the eye perpendicular to the cornea) and the visual axis (axis that intercepts the fixation point and fovea).^39^ In other words, angular misalignment between the eye and HMD lenses, as quantified by Δβ, can affect image quality. To validate this hypothesis, we performed optical bench measurements of the modulation transfer function (MTF) on the evaluated Meta Quest 2 HMD using an eye rotation geometry^22^^,^^41^ with additional rotation equal to an estimated angle kappa of 4 deg. It is shown in Fig. 8(a), on the right eyepiece, that the MTF measured at an azimuth angle of −9  deg (left, nasal rotation) is superior to the result at 9 deg (right, temporal rotation).

For group 2 experiments, Fig. 5 shows that the monocular CSRs of the left and right eyes are not substantially different. This can be illustrated by Figs. 7(c) and 7(d) that Δd and Δβ individually favor temporal and nasal eye rotation, respectively. Therefore, Δd and Δβ compensate for the effect of each other. In the MTF measurement shown in Fig. 8(b), with ΔIPD of −1  cm by shifting the camera lateral position by −5  mm, the temporal eye rotation (e.g., right eye gazing right, yellow curve) shows slightly higher MTF than the nasal eye rotation. However, the difference may not be substantial to be visualized in the ${\mathrm{CSR}}_{\mathrm{GL}}$ plots in the logarithm scale in Fig. 5, which can be potentially mitigated by increasing the number of group 2 participants in future work. Compared with CSRs measured using the appropriate IPD setting in Fig. 4, participants with smaller IPD than the HMD slightly enhance the contrast sensitivity for temporal eye rotation (e.g., right eye gazing toward right). A similar trend is shown in the MTF measurement in Figs. 8(a) and 8(b).

For group 3 experiments, the perceptual experiment shows much higher ${\mathrm{CSR}}_{\mathrm{GL}}$ for the nasal eye rotation (e.g., the right eye gazing toward left). This is consistent with the MTF measurements shown in Fig. 8(c) with ΔIPD of 1 cm. The interocular discrepancy in CSRs can be explained by Figs. 7(c) and 7(e) with both Δd and Δβ favor the nasal eye rotation. Smaller values of Δd and Δβ indicate that (1) IPD misalignment is minimized and (2) the optical axis of the HMD lens aligns with the pupillary axis of the eye, resulting in superior image quality on the HMD for nasal eye rotation.

## 4 Conclusion

We develop a WebXR test platform to evaluate monocular and binocular contrast perception on a VR HMD for human participants with various IPDs compared with the physical IPD setting of the HMD. For monocular perception, CSR decreases at the periphery of display FoV. It is illustrated that the interocular contrast sensitivity variation is associated with the shift of visual spot on the HMD eyepiece, which is determined by the participant and HMD’s IPD setting and gaze location (Δd). Besides, monocular vision is adjusted by the angle between the optical axes of the eye and HMD eyepiece lens, namely, Δβ. For participant with smaller IPD than the HMD, Δd and Δβ compensate each other resulting in similar CSF interocularly. On the other hand, for participant with larger IPD than the HMD optics, both Δd and Δβ favor nasal eye rotation leading to substantial difference in CSRs between the left and right eyes. Binocular vision is dominated by the eye with superior image quality. Note that the results presented in this work should only be applied to the evaluated VR HMD (Meta Quest 2) or other HMDs with very similar optical and display designs. On the other hand, the test platform and perceptual experimental method are generalizable to other VR HMDs with WebXR compatibility.

Limitations of this work include variations of visual capability among human participants, random error when determining the threshold contrast during the experiments, and a limited number of evaluated HMDs. In addition, unbalanced vision between the left and right eyes (e.g., dominant eye or amblyopia) may affect the monocular and binocular contrast perception results. To address these limitations, in future work, adding more participants can potentially reduce the statistical error. Binocular contrast perceptual performance may be compared with different HMDs to evaluate the impact of display and optics design on contrast sensitivity. Implementing a staircase yes–no detection method in the experiments can potentially minimize the participant input to reduce the random error.^36^ Finally, sophisticated binocular summation models^24^^,^^27^^,^^42^ may need to be investigated to bridge the gap between monocular and binocular contrast perception.

## Data Availability

This paper does not have associated code. Contrast sensitivity data from an individual human participant cannot be provided per FDA IRB protocol 2022-CDRH-038.

## References

[1] Hartmann T. Fox J., “Entertainment in virtual reality and beyond: the influence of embodiment, co-location, and cognitive distancing on users’ entertainment experience,” in The Oxford Handbook of Entertainment Theory, Vorderer P. Klimmt C., Eds., Oxford Academic (2021).

[2] Kamińska D. et al., “Virtual reality and its applications in education: survey,” Information 10(10), 318 (2019). 10.3390/info10100318

[3] Verhey J. T. et al., “Virtual, augmented, and mixed reality applications in orthopedic surgery,” Int. J. Med. Rob. Comput. Assist. Surg. 16(2), e2067 (2020). 10.1002/rcs.2067 31867864

[r4] Eastgate R. et al., “Modified virtual reality technology for treatment of amblyopia,” Eye 20(3), 370–374 (2006). 12ZYAS 0950-222X 10.1038/sj.eye.6701882 15832182

[5] Laver K. E. et al., “Virtual reality for stroke rehabilitation,” Cochr. Database Syst. Rev. 11(11), CD008349 (2017). 10.1002/14651858.CD008349.pub4 PMC6485957 29156493

[6] Sutherland J. et al., “Applying modern virtual and augmented reality technologies to medical images and models,” J. Digital Imaging 32, 38–53 (2019). JDIMEW 10.1007/s10278-018-0122-7 PMC6382635 30215180

[7] Pires F. Costa C. Dias P., “On the use of virtual reality for medical imaging visualization,” J. Digital Imaging 34, 1034–1048 (2021). JDIMEW 10.1007/s10278-021-00480-z PMC8455774 34327628

[8] Boedecker C. et al., “Using virtual 3D-models in surgical planning: workflow of an immersive virtual reality application in liver surgery,” Langenbeck’s Arch. Surg. 406, 911–915 (2021). 10.1007/s00423-021-02127-7 33710462 PMC8106601

[9] Xiao S. et al., “Randomized controlled trial of a dichoptic digital therapeutic for amblyopia,” Ophthalmology 129(1), 77–85 (2022). OPANEW 0743-751X 10.1016/j.ophtha.2021.09.001 34534556

[r10] Coco-Martin M. B. et al., “The potential of virtual reality for inducing neuroplasticity in children with amblyopia,” J. Ophthalmol. 2020, 7067846 (2020). 10.1155/2020/7067846 32676202 PMC7341422

[11] Žiak P. et al., “Amblyopia treatment of adults with dichoptic training using the virtual reality Oculus Rift head mounted display: preliminary results,” BMC Ophthalmol. 17(105), 1–8 (2017). 10.1186/s12886-017-0501-8 28659140 PMC5490155

[12] Pourmand A. et al., “Virtual reality as a clinical tool for pain management,” Curr. Pain Headache Rep. 22(53), 1–6 (2018). 10.1007/s11916-018-0708-2 29904806

[13] Beams R. et al., “Evaluation challenges for the application of extended reality devices in medicine,” J. Digital Imaging 35(5), 1409–1418 (2022). JDIMEW 10.1007/s10278-022-00622-x PMC9582055 35469355

[14] Johnson M. et al., “Quantifying the optical and rendering pipeline contributions to spatial resolution in augmented reality displays,” J. Soc. Inf. Disp. 32(8), 555–567 (2024). JSIDE8 0734-1768 10.1002/jsid.1297

[15] Wang J. et al., “Omnidirectional virtual visual acuity: a user-centric visual clarity metric for virtual reality head-mounted displays and environments,” IEEE Trans. Vis. Comput. Graphics 30, 2033–2043 (2024). 10.1109/TVCG.2024.3372127 38437113

[16] Patney A. et al., “Towards foveated rendering for gaze-tracked virtual reality,” ACM Trans. Graphics 35(6), 1–12 (2016). 10.1145/2980179.2980246

[17] Albert R. et al., “Latency requirements for foveated rendering in virtual reality,” ACM Trans. Appl. Percept. 14(4), 1–13 (2017). 10.1145/3127589

[18] Beams R. et al., “Angular dependence of the spatial resolution in virtual reality displays,” in IEEE Conf. Virtual Reality and 3D User Interfaces (VR), IEEE, pp. 836–841, (2020).

[19] Zhao C. Beams R. Badano A., “Radially variant contrast measurement in virtual reality headsets using circular concentric ring patterns,” J. Soc. Inf. Disp. 31(5), 387–397 (2023). JSIDE8 0734-1768 10.1002/jsid.1208

[20] Aizenman A. M. et al., “The statistics of eye movements and binocular disparities during VR gaming: implications for headset design,” ACM Trans. Graphics 42(1), 1–15 (2023). ATGRDF 0730-0301 10.1145/3549529 PMC10139447 37122317

[21] Martn S. et al., “Evaluation of a virtual reality implementation of a binocular imbalance test,” PloS One 15(8), e0238047 (2020). POLNCL 1932-6203 10.1371/journal.pone.0238047 32822405 PMC7446887

[22] IEC 63145-20-20:2019, “Eyewear display part 20-20: fundamental measurement methods image quality,” International Electrotechnical Commission, Geneva, CH (2019).

[23] “Information display measurements standard,” Society of Information Display (2023).

[24] Ding J. Klein S. A. Levi D. M., “Binocular combination of phase and contrast explained by a gain-control and gain-enhancement model,” J. Vision 13(2), 13–13 (2013).1534-7362 10.1167/13.2.13 PMC4521337 23397038

[r25] Ding J. Levi D. M., “A unified model for binocular fusion and depth perception,” Vision Res. 180, 11–36 (2021). 10.1016/j.visres.2020.11.009 33359897 PMC7856272

[r26] Ding J. Levi D. M., “Binocular combination of luminance profiles,” J. Vision 17(13), 4–4 (2017).1534-7362 10.1167/17.13.4 PMC5780634 29098293

[r27] Ding J. Sperling G., “A gain-control theory of binocular combination,” Proc. Natl. Acad. Sci. U. S. A. 103(4), 1141–1146 (2006). 10.1073/pnas.0509629103 16410354 PMC1347993

[r28] Huang C.-B. et al., “Contrast and phase combination in binocular vision,” PloS One 5(12), e15075 (2010). POLNCL 1932-6203 10.1371/journal.pone.0015075 21151558 PMC3000330

[r29] Freeman A. W., “Multistage model for binocular rivalry,” J. Neurophysiol. 94(6), 4412–4420 (2005). JONEA4 0022-3077 10.1152/jn.00557.2005 16148271

[30] Baker D. H. et al., “Nonlinearities in the binocular combination of luminance and contrast,” Vision Res. 56(1), 1–9 (2012). 10.1016/j.visres.2012.01.008 22289645

[31] Baker D. H. et al., “Binocular summation revisited: beyond √2,” Psychol. Bull. 144(11), 1186 (2018). PSBUAI 0033-2909 10.1037/bul0000163 30102058 PMC6195301

[32] IEC 63145-20-10:2019, “Eyewear display part 20-10: fundamental measurement methods—optical properties,” International Electrotechnical Commission, Geneva, CH (2019).

[33] Winters D. et al., “82-3: optical quality requirements for accurate MTF/CTF measurements on near-eye displays,” SID Symp. Digest Tech. Pap. 55, 1147–1150 (2024). DTPSDS 0097-966X 10.1002/sdtp.17742

[34] Barten P. G., Contrast Sensitivity of the Human Eye and Its Effects on Image Quality, SPIE Press, Bellingham, Washington (1999).

[35] Hoffman D. M. et al., “Vergence–accommodation conflicts hinder visual performance and cause visual fatigue,” J. Vision 8(3), 33–33 (2008).1534-7362 10.1167/8.3.33 PMC2879326 18484839

[36] Rousson J. et al., “Contrast sensitivity function in stereoscopic viewing of Gabor patches on a medical polarized three-dimensional stereoscopic display,” J. Electron. Imaging 25(2), 023014–023014 (2016). JEIME5 1017-9909 10.1117/1.JEI.25.2.023014

[37] Wang M. et al., “The effect of interocular contrast differences on the appearance of augmented reality imagery,” ACM Trans. Appl. Percept., 21(1), 1–23 (2023). 10.1145/3617684

[38] Wang M. et al., “16-1: a model for the appearance of interocular colorimetric differences in binocular XR displays,” SID Symp. Digest Tech. Pap. 55, 177–181 (2024). DTPSDS 0097-966X 10.1002/sdtp.17483

[39] Moshirfar M. Hoggan R. N. Muthappan V., “Angle kappa and its importance in refractive surgery,” Oman J. Ophthalmol. 6(3), 151 (2013). 10.4103/0974-620X.122268 24379548 PMC3872563

[40] Basmak H. et al., “Measurement of angle kappa with synoptophore and Orbscan II in a normal population,” J. Refract. Surg. 23(5), 456–460 (2007). 10.3928/1081-597X-20070501-06 17523505

[41] Zhao C. et al., “Integrating eye rotation and contrast sensitivity into image quality evaluation of virtual reality head-mounted displays,” Opt. Express 32(14), 24968–24984 (2024). OPEXFF 1094-4087 10.1364/OE.527660 39538921

[42] Blake R. Wilson H., “Binocular vision,” Vision Res. 51(7), 754–770 (2011). 10.1016/j.visres.2010.10.009 20951722 PMC3050089

